# Energy-Efficient Power Allocation and Relay Selection Schemes for Relay-Assisted D2D Communications in 5G Wireless Networks

**DOI:** 10.3390/s18092865

**Published:** 2018-08-30

**Authors:** Md Arifur Rahman, YoungDoo Lee, Insoo Koo

**Affiliations:** School of Electrical Engineering, University of Ulsan, 93-Daehak-ro, Namgu, Ulsan 44610, Korea, rassel.aece@gmail.com (M.A.R.); leeyd1004@naver.com (Y.L.)

**Keywords:** device-to-device communications, Internet of Things, energy efficiency, relay selection, power allocation, particle swarm optimization

## Abstract

Device-to-device (D2D) communications allows user equipment (UE) that are in close proximity to communicate with each other directly without using a base station. Relay-assisted D2D (RA-D2D) communications in 5G networks can be applied to support long-distance users and to improve energy efficiency (EE) of the networks. In this paper, we first establish a multi-relay system model where the D2D UEs can communicate with each other by reusing only one cellular uplink resource. Then, we apply an adaptive neuro-fuzzy inference system (ANFIS) architecture to select the best D2D relay to forward D2D source information to the expected D2D destination. Efficient power allocation (PA) in the D2D source and the D2D relay are critical problems for operating such networks, since the data rate of the cellular uplink and the maximum transmission power of the system need to be satisfied. As is known, 5G wireless networks also aim for low energy consumption to better implement the Internet of Things (IoT). Consequently, in this paper, we also formulate a problem to find the optimal solutions for PA of the D2D source and the D2D relay in terms of maximizing the EE of RA-D2D communications to support applications in the emerging IoT. To solve the PA problems of RA-D2D communications, a particle swarm optimization algorithm is employed to maximize the EE of the RA-D2D communications while satisfying the transmission power constraints of the D2D users, minimum data rate of cellular uplink, and minimum signal-to-interference-plus-noise-ratio requirements of the D2D users. Simulation results reveal that the proposed relay selection and PA methods significantly improve EE more than existing schemes.

## 1. Introduction

Future fifth-generation (5G) wireless networks are expected to be extremely heterogeneous in their architectures, allowing the coexistence of femtocell, microcell, and device-to-device (D2D) communications [1,2,3,4]. The development of conventional wireless networks into 5G wireless networks is driven by an incredible growth in wireless mobile devices, high data rates, high energy efficiency (EE), low latency, and improved quality of service [5]. To address these challenges, 5G wireless networks rely on some key technologies, such as full-duplex operation by users, network diversification, and D2D communications. Unlike the conventional cellular wireless communications networks, D2D devices can interconnect with each other directly, without involving a cellular base station (BS). Some advantages can be achieved through D2D communications, such as spectrum efficiency, overall system throughput, EE, extension of the coverage area, and reduction of transmission delay between devices. The D2D users can communicate with each other by utilizing licensed or unlicensed spectra. In D2D-enabled cellular networks, the spectrum-sharing mechanism is mainly divided into two modes: overlay mode and underlay mode. In overlay mode, D2D users are unable to use the spectrum while cellular users (CUs) utilize them, or D2D user equipment (UEs) are allocated a dedicated spectrum, which can effectively avoid interference between D2D UEs and cellular communications. In underlay mode, the D2D users can simultaneously use the resources of cellular networks in a reasonable way. However, there may be serious interference between D2D users and cellular users due to the sharing of the same resources. Cooperative communications (CC) facilitate collaboration among D2D UEs, and it may provide a smart solution for efficient utilization of cellular uplink spectrum resources. In CC, the D2D source exploits a cooperative D2D relay to transmit D2D source information to the D2D destination. CC can also reduce interference by reducing the transmission power of the D2D UEs. Relay cooperation in RA-D2D communications not only reduces the transmission power of the D2D UEs in order to protect CUs, but it can also enhance EE in the networks. In underlay mode, the important research challenge for RA-D2D communications is to maintain the interference via relay selection (RS) and power allocation (PA). Consequently, energy-efficient RS and PA solutions for RA-D2D communications are useful ways to limit the interference to an acceptable range in order to protect the communications of cellular users while maximizing the EE of RA-D2D communications.

### 1.1. Literature Review

D2D communications mainly aim to maximize the throughput or spectral efficiency of networks. Joint RA and a low complexity RS strategy for D2D communications-enabled cellular networks were studied in [6], where D2D UEs can act as a source and potential relay to support other D2D UEs. A bargaining game for the RA problem and a simple heuristic algorithm for RS were proposed to maximize the data rate of the networks. Similarly, joint RS and RA in cooperative D2D communications were proposed by Wang et al. [7], where multiple potential relays are selected to support long-distance D2D UEs, and a cooperative game was also formulated to maximize each cooperator’s data rate. However, some important performance metrics of D2D communications in 5G networks (i.e., EE and energy consumption) are out of scope in their works. Therefore, an energy-efficient PA for RA-D2D communications in 5G networks was studied in [8], where the EE of the networks is maximized by optimal power allocation to the D2D transmitter and the D2D relay by transforming a nonlinear fractional problem into nonlinear parameter problem. Even though, the EE of RA-D2D communications in 5G networks was studied, a multi-relay system model and RS mechanism to forward the D2D source information to the D2D destination was outside the scope in their work. Joint resource block assignment and PA problems for D2D communications underlying a full-duplex cellular network were studied in [9], where a low complexity graph coloring-based resource management scheme was proposed to solve joint optimization problems in order to maximize the throughput of the network. Mode selection and RA problems for a D2D-enabled two-tier cellular network were proposed in [10], where a geometric vertex-based search approach to solve the PA problem of the resource reuse mode was proposed to maximize the sum rate of the network. However, in resource reuse mode, joint RS and PA to maximize the EE of D2D communications underlying cellular networks was not studied by the authors, which is essential for 5G wireless networks.

In [11], power coordination, RS, and mode selection problems were studied in which relay-assisted D2D communications and local route-based D2D communications were proposed to enhance the traffic offloading capacity of cellular-assisted D2D communications. In full-duplex cellular networks, RA for physical layer security was studied in [12], where bipartite graph-based joint resource block assignment and PA were proposed to maximize the secrecy capacity of the network. In [13], an overview of D2D communications in cellular networks and some optimization approaches were studied to solve the RA problem of the network. However, commonly used objective functions (e.g., EE, battery life, capacity, interference management, and network connectivity) in the D2D paradigm were not evaluated in the paper. Joint channel allocation and power control for underlay D2D transmission was studied in [14], where a centralized graph-theoretical channel allocation approach for D2D and cellular users was proposed to maximize the utility of the network. The subsequent power control of the network was modeled as a game, and a multi-agent Q-learning strategy was used to solve the formulated game to maximize utility of the network. Efficient power-control approach for half-duplex relay-based D2D networks under sum power constraints was studied in [15], where the authors gave priority to the cellular links by satisfying their minimum data rate requirements in order to maximize the achievable data rate of local D2D users. Under the sum power constraints of the network, a closed form solution was proposed to allocate optimal power to the BS, the D2D transmitter, and the power-splitting factor at the D2D transmitter. PA for full-duplex relay–based D2D underlying cellular networks was studied by Zhang et al. [16], where a closed-form solution was provided to maximize the data rate of D2D communications. User association scheme with the presence of user mobility was studied in [17], where a mobility-aware user association (UA) strategy is proposed to overcome the problems of conventional received power-based UA of 5G mmWave networks. The computational complexity problem of exhaustive search was solved by distributed manner and the overall achievable data load of the networks is maximized based on proper UA strategies.

In summary, existing works on D2D communications have generally studied RS, RA, PA, mode-selection, and UA problems either separately or jointly, to maximize the data rate, throughput, sum rate, spectral efficiency, traffic offloading capacity, total secrecy capacity, utility, and acheivable data load of the networks [6,7,8,9,10,11,13,14,15,16,17]. To the best of our knowledge, a joint solution for RS and PA, maximizing the EE of RA-D2D communications in 5G networks to support applications in the Internet of Things (IoT), is still an open problem.

### 1.2. Paper Contributions

In this paper, we propose RA-D2D communications that incorporate RS and PA problems in a two-layered underlying cellular network where D2D UEs use the resources simultaneously with cellular users in a cellular uplink mode. We study the circumstances in which a D2D user source transmits information to a D2D user destination with the aid of a selected D2D relay. Optimal D2D RS is a complex evaluation process, and requires all information of the D2D relays. Fuzzy logic and approximate reasoning can simplify this complex optimal D2D RS process. Considering that the adaptive neuro-fuzzy inference system (ANFIS) architecture can learn the behavior of the system in a distributed manner [18], we then propose an ANFIS architecture-based RS scheme to select the best D2D relay to forward the D2D source information to the D2D destination. We also propose a particle swarm optimization (PSO) algorithm to allocate transmission power to the D2D source and the selected D2D relay in order to maximize the EE of the network. The comprehensive contributions of this paper can be summarized as follows

- We propose an ANFIS architecture-based energy-efficient D2D RS algorithm in which the joint received signal-to-interference-plus-noise ratio (SINR) at the D2D destination, and the received SINR at the BS are utilized as inputs to the proposed ANFIS architecture. We show that our proposed RS algorithm can effectively select the D2D relay without degrading performance with an exhaustive search-based RS scheme.
- We investigate the PA problems of the D2D source and the D2D relay in order to maximize the EE of RA-D2D communications to support the emerging applications in the IoT. Considering that the PSO algorithm has been shown to be an efficient method of solving PA problems in wireless communications [19,20,21,22], we then propose a PSO algorithm-based PA scheme to assign transmission power to the D2D source and the D2D relay to maximize the EE of RA-D2D communications.
- We verify through simulation results that the proposed ANFIS architecture-based RS and PSO-based PA schemes significantly improve EE performance more than conventional schemes, and show near optimal EE performance to that of exhaustive search-based RS and PA schemes.

The remainder of this paper is organized as follows. The system model and the problem formulation of the proposed scheme are shown in Section 2. The ANFIS architecture for RS, and the proposed PSO algorithm for PA, are briefly described in Section 3 and Section 4, respectively. Simulation results of the proposed scheme and all compared schemes are presented in Section 5. Finally, we conclude this paper with future research directions in Section 6.

## 2. System Model

**Figure 1:**
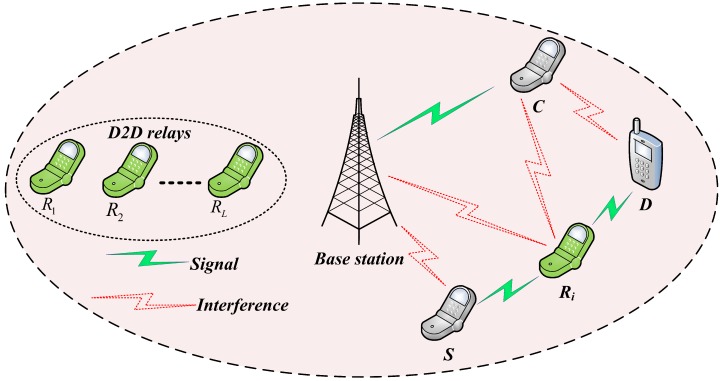
System model.

As shown in Figure 1 we consider a two-layer cellular network which coexists with one cellular base station, one cellular user (C), one D2D source (S), one D2D destination (D) and the D2D relay node are denoted as $R_{i}$, where i=(1,2,3,…,L). The considered system model is a frequency division duplex network and each RA-D2D links reuse only one cellular uplink resource [8] assigned to the cellular user. In D2D-enabled cellular networks, the spectrum-sharing mechanism is mainly divided into two modes: overlay mode and underlay mode. In the paper, we consider an underlay mode of spectrum-sharing where the D2D users can simultaneously utilize the resources of cellular networks in uplink. Therefore, the D2D relay-nodes together with the D2D destination will be interfered by the cellular user. Moreover, the base station will also be interfered by the D2D source as well as D2D relay in the process of information transmission. In the paper, we also assume that the uplink resoucre is already allocated to the cellular user, and it is also utilized by the RA-D2D links in an underlay mode where the D2D users can simultaneously use the resources of cellular networks in a reasonable way. However, there may exist serious interference between D2D users and cellular user due to the sharing of same resources. In this circumstance, reasonable mechanism of interference management and power control are needed to overcome the mutual interference into an acceptable level. Therefore, we apply an ANFIS architecture to select the best D2D relay based on considering two important parameters: link SINR of RA-D2D communications and the received SINR at the BS.

**Figure 2:**
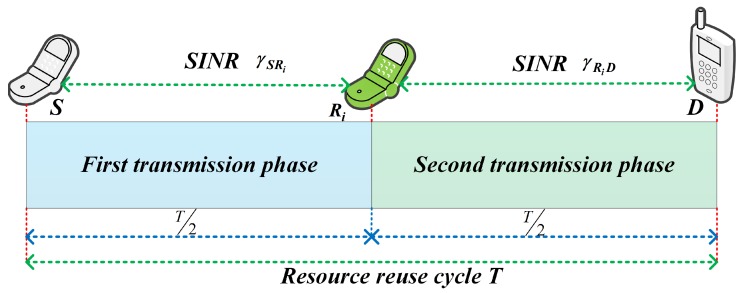
Frame structure of the proposed scheme.

We assume a centralized method where the channel state information (CSI) for all communications links is supposed to be acquirable, and the interference between D2D communications and cellular users can be computed and synchronized precisely. The selected *i*-th D2D relay under the proposed ANFIS architecture forwards the information of the D2D source to the D2D destination. The *i*-th D2D relay will interfere with a cellular user when it receives information from the D2D source. We also assume that each D2D relay operates in half-duplex mode, and a resource reuse cycle is separated into two hops. Information transmission from the D2D source to the D2D destination via D2D relay can be divided into two phases, as shown in Figure 2. In the first $\frac{T}{2}$ phase, the D2D source transmits its information while the D2D relays will only listen; and in the second $\frac{T}{2}$ phase, the selected D2D relay will forward the information to the D2D destination. In this paper, it is assumed that the D2D destination only receives information in the second $\frac{T}{2}$ phase of the total transmission cycle. The main symbols used in this paper are listed in Table 1.

### 2.1. Problem Formulation and Analysis

Let us denote $P_{S}$, $P_{C}$, and $P_{R_{i}}$ as transmission power of the D2D source, of the cellular user, and of the *i*-th D2D relay to forward the D2D source information to the D2D destination, respectively. In this paper, the following transmit power constraint is considered in order to maximize the EE of RA-D2D communications:

(1) $$P_{S}\;and\;P_{R_{i}}\leq P_{Max}$$

The SINR from the D2D source to the D2D relay and from the D2D relay to the D2D destination, respectively, can be expressed as

(2) $$\gamma_{SR_{i}}=\frac{P_{S}h_{SR_{i}}}{P_{C}h_{CR_{i}}+N_{0}}$$

(3) $$\gamma_{R_{i}D}=\frac{P_{R_{i}}h_{R_{i}D}}{P_{C}h_{CD}+N_{0}}$$

In cooperative communications, relaying protocols are generally classified as either amplify-and-forward (AF) or decode-and-forward (DF), according to the information processing technique at the D2D relay [23]. In the considered system model, the D2D relay will be affected by the cellular user transmission when the D2D relay receives the information from the D2D source. The interference, together with the information, will be amplified if the AF protocol is employed at the D2D relay. Consequently, the D2D destination will have a low SINR when the D2D relay forwards the information. To avoid that problem in the AF protocol, the DF protocol is employed in this paper to avoid the interference before forwarding the actual received information. The D2D destination only accepts information in the second time slot. Therefore, the joint received SINR at the D2D destination can be expressed as

(4) $$\gamma_{SR_{i}D}=\min\left\{\gamma_{SR_{i}},\gamma_{R_{i}D}\right\}$$

The data rate of the RA-D2D links is defined in [8] as follows

(5) $${ℜ}_{SR_{i}D}=\frac{1}{2}W\;\log_{2}\left(1+\gamma_{SR_{i}D}\right)$$

where *W* is the bandwidth and the bandwidth factor $\frac{1}{2}$ specifies that RA-D2D communications will consume resources in two phases as compared to direct transmission scheme. Upon receiving the D2D source information, the BS will receive interference from the transmissions of the D2D source and the D2D relay due to the sharing of spectrum resources with RA-D2D links. The interference at the BS due to the D2D source and the D2D relay transmissions is divided into two phases. Therefore, the received SINR at the BS can be expressed as

(6) $$\gamma_{CB}=\left\{\begin{array}{c} \frac{P_{C}h_{CB}}{P_{S}h_{SB}+N_{0}},\;\;the\;first\;\;\frac{T}{2}\;transmission\;phase\\ \frac{P_{C}h_{CB}}{P_{R_{i}}h_{R_{i}B}+N_{0}},\;\;the\;2nd\;\;\frac{T}{2}\;transmission\;phase\;\; \end{array}\right.$$

The data rate at the BS from the cellular user transmission can be expressed as

(7) $${ℜ}_{CB}=W\;\log_{2}\left(1+\gamma_{CB}\right)$$

We define the EE in the PA of the D2D source and the D2D relay as the fraction between the data rate and the total power consumption. The EE of the RA-D2D communications can be expressed as

(8) $$E_{SR_{i}D}=\frac{{ℜ}_{SR_{i}D}}{P_{Total}}$$

The power consumed by both D2D source and D2D relay transmissions can be expressed as

(9) $$P_{Total}=\frac{1}{2}\left(\frac{P_{S}}{\lambda }+\frac{P_{R_{i}}}{\lambda }+4P_{Cir}\right)$$

where λ represents the power amplifier efficiency, i.e., 0<λ<1 . The circuit power of the system can be generated from two different time slot transmissions in one full transmission cycle: (1) the circuit power at both D2D source and the *i*-th D2D relay sides in the first $\frac{T}{2}$ transmission phase, and (2) the circuit power at both *i*-th D2D relay and the D2D destination in the second $\frac{T}{2}$ transmission phase. The transmission power of the D2D source and the *i*-th D2D relay, along with the circuit power for both D2D source and D2D destination, will be consumed during half the transmission cycle. Therefore, total transmission power of the system will be multiplied by a factor of $\frac{1}{2}$.

Our objective in this work is to maximize EE by selecting the best relay, and allocating optimal power to the D2D source and to the selected D2D relay while satisfying the system constraints. Therefore, we have divided the optimization problem into two subproblems: an RS subproblem, and a PA subproblem. To solve the RS subproblem, we construct a mapping model between the D2D RS parameters and RS factor, denoted by $\theta_{F}$ through the proposed ANFIS architecture. The proposed ANFIS architecture to solve the RS subproblem is discussed in the next section. The transmission PA subproblem to maximize EE can be expressed as

(10) $$P_{S}^{*},P_{R_{i}}^{*}=\underset{P_{S},\;P_{R_{i}}}{\arg\max}\left(\frac{\frac{1}{2}W\;\log_{2}\left(1+\gamma_{SR_{i}D}\right)}{\frac{1}{2}\left(\frac{P_{S}}{\lambda }+\frac{P_{R_{i}}}{\lambda }+4P_{Cir}\right)}\right)$$

Subjected to:

(11a) $$\begin{array}{c} 0\leq P_{S}\;and\;P_{R_{i}}\leq P_{Max} \end{array}$$

(11b) $$\begin{array}{c} \frac{P_{S}h_{SR_{i}}}{P_{C}h_{CR_{i}}+N_{0}}\geq \gamma_{R}^{Min} \end{array}$$

(11c) $$\begin{array}{c} \frac{P_{R_{i}}h_{R_{i}D}}{P_{C}h_{CD}+N_{0}}\geq \gamma_{D}^{Min} \end{array}$$

(11d) $$\begin{array}{c} {ℜ}_{CB}\geq \;{ℜ}_{C}^{Min} \end{array}$$

where (11a) represents the maximum power constraints for the D2D users, while (11b)–(11d) are minimum SINR requirements and the minimum data rate of the cellular user, respectively. In this paper, we use resource uplink underlay mode and the resources are shared by the D2D UEs. Therefore, a minimum rate constraint is equivalent to a minimum SINR constraint.

### 2.2. Transmission Power at Cellular User

As inferred from Equation (10), the objective function (the EE) is a reduction function with respect to the cellular power. Consequently, if we want to maximize the EE of RA-D2D communications, $P_{C}$ needs to be minimized, which means

(12) $${ℜ}_{C}^{Min}=\left\{\begin{array}{c} W\log_{2}\left(1+\frac{P_{C}h_{CB}}{P_{S}h_{SB}+N_{0}}\right),\;\;the\;first\;\;\frac{T}{2}\;transmission\;phase\;\\ W\log_{2}\left(1+\frac{P_{C}h_{CB}}{P_{R_{i}}h_{R_{i}B}+N_{0}}\right),\;\;the\;\;2nd\;\;\frac{T}{2}\;\;transmission\;phase \end{array}\right.$$

To simplify the transmission power of the cellular user we always allow the potential maximum interference with the BS during D2D source and D2D relay transmissions when calculating optimal transmission power of the cellular user. We can thus rewrite Equation (12) as

(13) $${ℜ}_{C}^{Min}=W\log_{2}\left(1+\frac{P_{C}^{Opt}h_{CB}}{\max\left(P_{Max}h_{SB},\left(P_{Max}\underset{i=\left\{1,2,3,\ldots ,L\right\}}{\max\left(h_{R_{i}B}\right)}\right)+N_{0}\right)}\right)$$

The optimal transmission power at the cellular user can be obtained by solving Equation (13) as follows:

(14) PCOpt=2ℜCMinW−1hCBmaxPMaxhSB,PMaxmaxhRiBi=1,2,3,…,L+N0

## 3. Proposed ANFIS Architecture for Relay Selection

**Figure 3:**
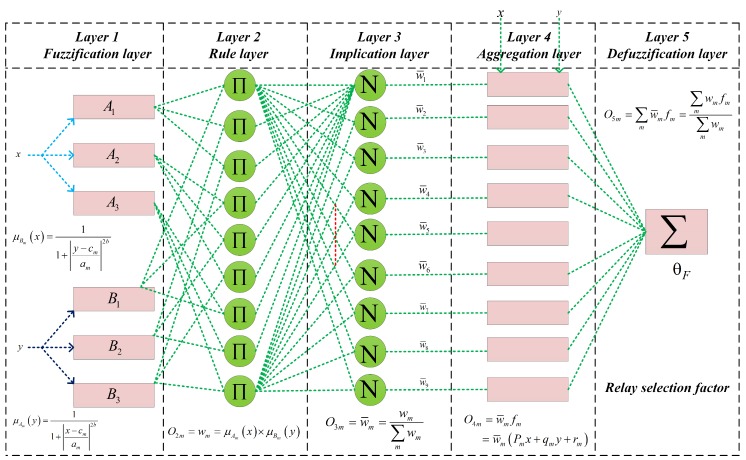
Proposed ANFIS architecture for relay selection.

The proposed ANFIS architecture is an adaptive network that uses supervised learning and which has a function like a model of the Takagi–Sugeno fuzzy inference system. We use two inputs *x* and *y* in our proposed ANFIS architecture, which are link SINR of the RA-D2D communications and SINR at the BS during D2D relay transmission. Figure 3 shows the ANFIS architecture which consists of five layers. The nodes in the layer 1 generate membership grades by using the fuzzification process of the fuzzy inference system. We also use three linguistic levels to fuzzify the input variables of the ANFIS, which makes nine different rules in the rule layer. The nodes in layer 2 produce the firing strengths, or the rule weight coefficient, whereas layer 3 calculates the qualified consequent membership functions (MFs) based on the firing strength from layer 2. The qualified consequent MFs from layer 3 are then aggregated to generate an overall output membership function in layer 4. Finally, after defuzzification, the output of the MFs are converted into crisp output values in layer 5.

### 3.1. ANFIS Architecture Description

Brief explanations of each layer of the proposed ANFIS architecture are as follows.

**Layer 1**: Each node in layer 1 adapts to a function parameter. The output from each node is a degree-of-membership value that is given by the input of the membership function. In the proposed scheme, we utilize a generalized bell membership function to fuzzify the input parameters. The membership functions can be expressed as

(15) $$\mu_{A_{m}}\left(x\right)=\frac{1}{1+{\left|\frac{x-c_{m}}{a_{m}}\right|}^{2b}}$$

(16) $$\begin{array}{c} O_{1,\;m}=\mu_{A_{m}}\left(x\right),\;\;\;\;\;\;\;m=1,2,3\\ O_{1,\;m}=\mu_{B_{m-3}}\left(y\right),\;\;\;\;m=4,5,6 \end{array}$$

where $\mu_{A_{m}}\left(x\right)$, $\mu_{B_{m}}\left(y\right)$, and $\left\{a_{m},b_{m},c_{m}\right\}$, respectively are the degree of membership functions for fuzzy sets $A_{m}$ and $B_{m}$ and the parameters of the membership which can change the shape of the membership functions. The parameters are referred to as the premise parameters. The fuzzy sets of the input parameters are represented as

(17) $$\begin{array}{c} A_{m}=\left\{A_{1},A_{2},A_{3}\right\}\\ B_{m}=\left\{B_{1},B_{2},B_{3}\right\} \end{array}$$

where $A_{m}$ and $B_{m}$ reflect the linguistic levels of the input variables. The linguistic level of the link SINR of RA-D2D communications and the received SINR at the base station are denoted as: $\left\{Low,Medium,High\right\}$ and $\left\{High,Medium,Low\right\}$, respectively.

**Layer 2**: The output node of this layer is the result of multiplying the incoming signal from layer 1. Each node in this layer is the firing strength for each rule. In layer 2, the T-norm operator with general performance (for example, the AND operator) is used to acquire the output. The output at layer 2 of the ANFIS architecture can be expressed as

(18) $$O_{2m}=w_{m}=\mu_{A_{m}}\left(x\right)\times \;\mu_{B_{m}}\left(y\right),\;\;\;\;m=\left\{1,2,3,4,5,6\right\}$$

**Layer 3**: Each node in this layer will calculate the ratio between the *m*-th rule’s firing strength and the sum of all the rules’ firing strengths. In this work, we consider this firing strength the normalized firing strength of the ANFIS, and the value can be expressed as

(19) $$O_{3m}=\bar{w_{m}}=\frac{w_{m}}{\sum_{m}w_{m}}$$

**Layer 4**: Every node in layer 4 is an additive node, which can be expressed as

(20) $$\begin{array}{c} O_{4m}={\bar{w}}_{m}f_{m}\\ \;\;\;\;\;\;\;\;={\bar{w}}_{m}\left(p_{m}x+q_{m}y+r_{m}\right) \end{array}$$

where ${\bar{w}}_{m}$ is the normalized firing strength from layer 3, and $p_{m}$, $q_{m}$, and $r_{m}$ are the consequent parameters of the ANFIS architecture.

**Layer 5**: The single node in layer 5 is a fixed or non-additive node that calculates the output as a summation of all incoming signals from the previous node. In this layer, we can decide the result based on the solution approaches of the RS optimization problem, as follows:

(21) $$O_{5m}=\sum_{m}{\bar{w}}_{m}f_{m}=\frac{\sum_{m}w_{m}f_{m}}{\sum_{m}w_{m}}$$

We constructed a mapping model between the D2D RS parameters and RS factor, denoted by $\theta_{F}$ through the proposed ANFIS architecture. The neuro fuzzy-based algorithm selects D2D relay that has the maximum EE of the RA-D2D communications. The RS method of the proposed ANFIS architecture can be expressed as

(22) $$\theta_{F}=\underset{i=\left\{1,2,3,\ldots ,L\right\}}{\arg\max}ℑ\;\left(\gamma_{SR_{i}D},\gamma_{CB}\right)$$

### 3.2. Training of ANFIS Architecture

**Figure 4:**
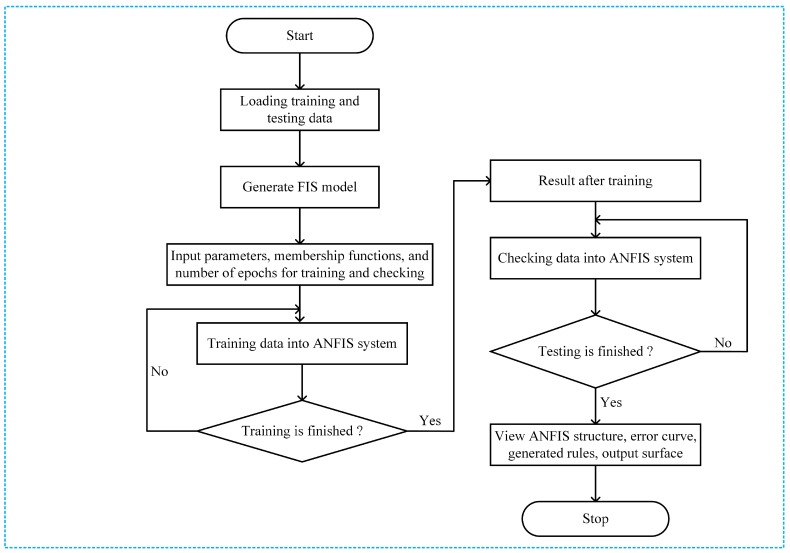
ANFIS training system.

The ANFIS system training procedure is summarized in Figure 4. The training procedure for the ANFIS starts by collecting a training data set (input/output data pairs) from the considered system model. We generate data by changing the D2D relay positions within transmission range of the D2D source in the network. The training data are a set of input and output vectors. The input vectors of the training data are comprised of two elements; the first column of the corresponding data set is related to the joint received SINR at the destination, and the second column is related to the received SINR at the BS during the second time slot. The output vector is related to the EE of the networks.

The training data set is used to obtain the premise parameters of the membership functions. ANFIS training begins by creating a set of suitable data to train the neuro-fuzzy system. The row of the matrix represents the amount of data sampled in the data set. We train the ANFIS architecture by using generalized bell membership functions for both inputs to fuzzify the inputs. The ANFIS training procedure begins by defining (1) the fuzzy sets, (2) the number of sets of each input variable, and (3) the shape of the membership function. All the training data pass through the neural network to adjust the input parameters, to find the relationship to the expected output, and to minimize the error while training the ANFIS. The output (the EE) of the networks does not require evaluating the ANFIS architecture. With the output from the ANFIS, and based on Equation (22), we select the best D2D relays to forward the D2D source information to the expected D2D destination. Once the ANFIS is trained, we can test the system against the testing data set to check the errors from training and testing the considered ANFIS architecture. In this paper, we use the root-mean-square-error (RMSE) function to calculate the training error. The RMSE function was defined in [18] as follows

(23) $$\mathrm{RMSE}=\sqrt{\frac{1}{N}\sum_{t=1}^{N}{\left(y_{t}-\tilde{y}\right)}^{2}}$$

where *N*, $y_{t}$, and $\tilde{y}$ are the total number of prediction, desired output, and predicted output, respectively. The ANFIS architecture’s structure information is in Table 2.

**Figure 5:**
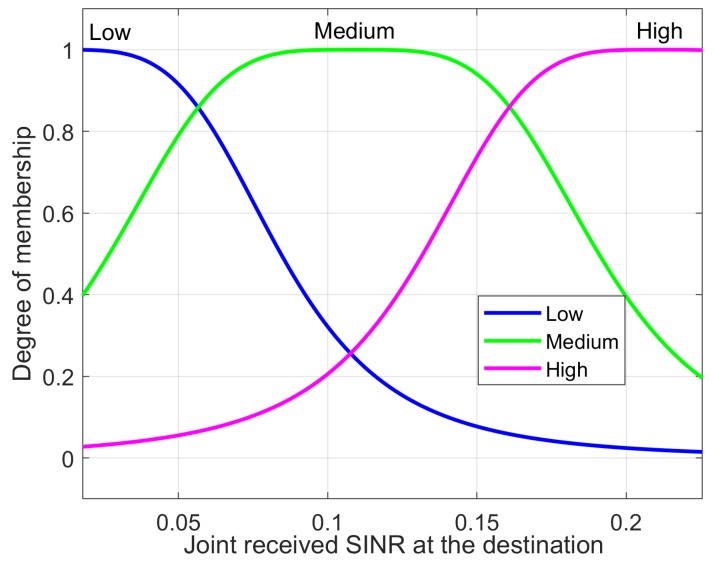
Input (joint received SINR at the destination) membership function.

**Figure 6:**
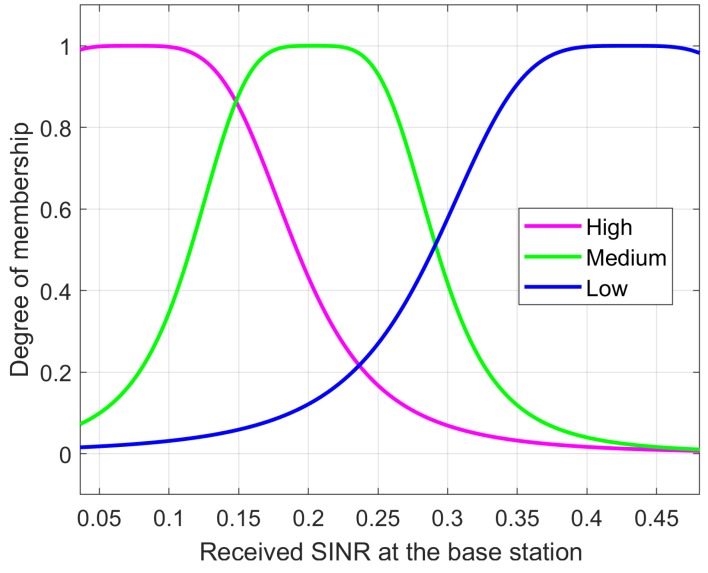
Input (received SINR at the BS) membership function.

**Figure 7:**
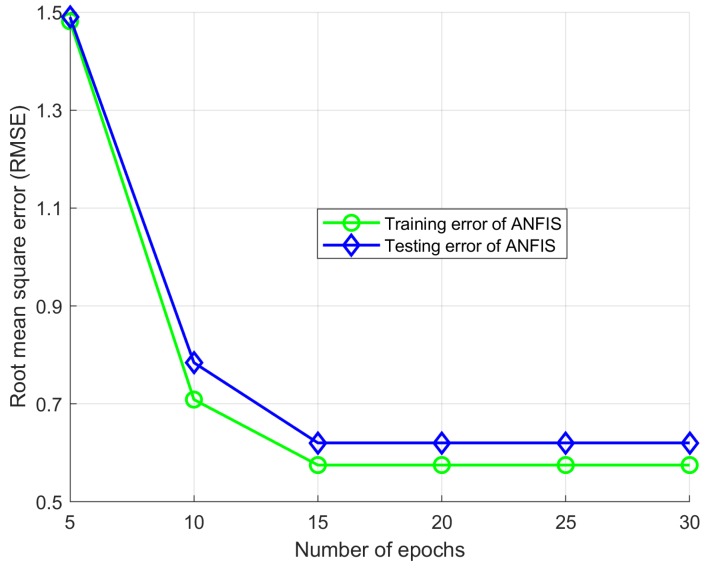
Training and testing errors according to the number of epochs.

**Figure 8:**
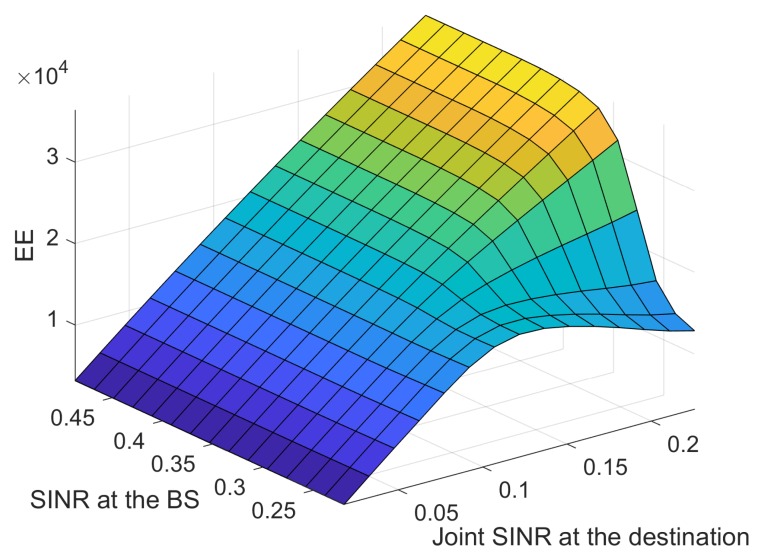
The surface viewer of ANFIS after training.

The MFs of two inputs are presented in Figure 5 and Figure 6. The difference in the RMSE between the desired and the modeled values is calculated for each trial with different epoch numbers, and the best structure is determined by selecting the lowest RMSE. Each data set used for training has its own maximum number epochs to prevent overfitting; this causes the predicted output to exceed its accuracy. The best number of epochs is selected by conducting numerous simulation runs. Overfitting was analyzed by observing Figure 7 where the training and testing error values are plotted against the number of epochs. To avoid the overfitting problem, we tested the ANFIS architecture by setting different values for epoch numbers. We determined the optimal epoch number with the lowest RMSE. The surface viewer of Figure 8 is used to validate the dependency of the outputs on two of the inputs.

## 4. Proposed PSO Algorithm for Power Allocation

The PA subproblem in this paper is changed into a problem of maximizing the EE of the network while satisfying the constraints of the network. We use a particle swarm optimization algorithm to allocate transmission powers to the D2D source and the selected D2D relay to maximize the EE of the network. The first phase of the PSO algorithm is to select the parameters that need to be optimized, and then to determine the lower and upper boundaries of the parameters while searching for the optimal solution to the problem. We define minimum transmission power of the D2D source and relay as the lower search boundary, and define the maximum transmission power of the D2D source and relay as the upper search boundary of the proposed PSO algorithm. The minimum transmission power for the D2D source and the D2D relay, respectively, can be calculated as

(24) $$P_{S}^{Min}=\frac{\gamma_{R}^{Min}N_{0}}{h_{SR_{i^{*}}}}\leq P_{S}\leq P_{Max}$$

(25) $$P_{{R_{i}}_{*}}^{Min}=\frac{\gamma_{D}^{Min}N_{0}}{h_{R_{i^{*}}D}}\leq P_{R_{i^{*}}}\leq P_{Max}$$

**Figure 9:**
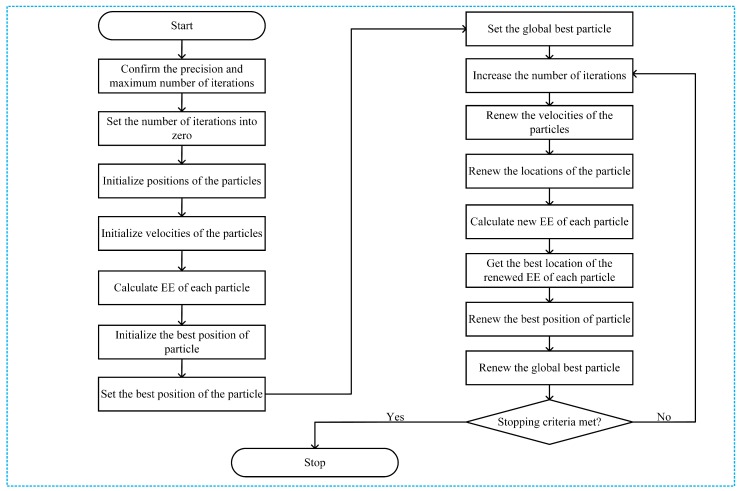
Flowchart of the proposed PSO algorithm for PA.

The operational procedure of the proposed PSO is shown in Figure 9, where the location of the *k*-th particle is set as the power allocated to the D2D source and the D2D relay. The location of the *k*-th particle $\left(k=1,2,3,\ldots ,q\right)$ is set as $P_{S_{k}}$, $P_{R_{i^{*},k}}$, where *q* denotes the number of particles in the particle swarm.

(26) $$z_{k}=\left\{P_{S_{k}},P_{R_{i^{*},k}}\right\}$$

We see in Equation (26) that the dimension of the particles in the proposed PSO algorithm is two. At the beginning of PSO, we initialize the positions and velocities of the particles randomly within the search boundary, and then, we calculate the fitness (the EE) of each particle. The first global best location is selected by choosing the maximum value of these initial positions. The velocities of the particles are updated according to the relative locations of the individual best and the global best locations, as follows:

(27) $$v_{k,Source}^{\left(w+1\right)}=\omega^{\left(w\right)}v_{k,Source}^{\left(w\right)}+l_{1}r_{1}\left(pb_{k}^{\left(w\right)}-z_{k}^{\left(w\right)}\right)+l_{2}r_{2}\left(gb^{\left(w\right)}-z_{k}^{\left(w\right)}\right)$$

(28) $$v_{k,\;Relay}^{\left(w+1\right)}=\omega^{\left(w\right)}v_{k,\;Relay}^{\left(w\right)}+l_{1}r_{1}\left(pb_{k}^{\left(w\right)}-z_{k}^{\left(w\right)}\right)+l_{2}r_{2}\left(gb^{\left(w\right)}-z_{k}^{\left(w\right)}\right)$$

(29) $$v_{k}^{\left(w\right)}=\left(v_{k,Source}^{\left(w\right)},v_{k,\;Relay}^{\left(w\right)}\right)$$

where $v_{k}^{\left(w\right)}$ is the velocity of the *k*-th particle and $z_{k}^{\left(w\right)}$ is the location of the *k*-th particle in the *w*-th iteration, respectively. In addition, $l_{1}$ and $l_{2}$ are scaling factors; $l_{1}$ and $l_{2}$ are the random numbers between 0 and 1. We denote $pb_{k}^{\left(w\right)}$ and $gb^{\left(w\right)}$ as the individual best location of the *k*-th particle and the global best location of all particles in the *w*-th iteration, respectively. We also denote ω as the inertial weight, and this inertial weight of the proposed PSO is determined in such a way that the particle leftover original sequence will be unaffected by the pulling $pb_{k}^{\left(w\right)}$ and $gb^{\left(w\right)}$, respectively. In this paper, the *w*-th iteration of the proposed PSO is given by

(30) $$\omega^{\left(w\right)}=\frac{\omega_{Start}-\left(\omega_{Start}-\omega_{End}\right)w^{2}}{I_{T}^{2}}$$

where $\omega_{Start}$ = 0.9, $\omega_{End}$ = 0.4, and $I_{T}$ is the maximum number of iterations.

The locations of the particles are accelerated as follows

(31) $$z_{k}^{\left(w+1\right)}=z_{k}^{\left(w\right)}+v_{k}^{\left(w+1\right)}$$

where $z_{k}^{\left(w\right)}$ is the location of the *k*-th particle in the *w*-th iteration. The EE of each particle is re-calculated and confidentially the global optimum fitness (EE) of the network are renewed. The process is repeated until meeting the stopping criteria. The proposed PSO allocation to maximize the EE described below as shown specifically in Algorithm 1.

**Algorithm 1:** Proposed PSO algorithm for PA.
**1**

**Start**

**2**
Confirm ξ and $I_{T}$
**3**

w=0

**4**
**Initialize** positions of the particles $z^{\left(w\right)}=\left\{\left(P_{S_{1}}^{\left(w\right)},P_{R_{i^{*},1}}^{\left(w\right)}\right),\ldots ,\left(P_{S_{q}}^{\left(w\right)},P_{R_{i^{*},q}}^{\left(w\right)}\right)\right\}$
**5**
**Initialize** velocities of the particles $v^{\left(w\right)}=\left(v_{1}^{\left(w\right)},v_{2}^{\left(w\right)},\ldots ,v_{q}^{\left(w\right)}\right)$
**6**
**Calculate** EE of each particle $EE_{k}^{\left(w\right)},\left(k=1,2,\ldots ,q\right)$
**7**
**Initialize** the best position of particle $pb_{k}^{\left(w\right)}=z_{k}^{\left(w\right)},\left(k=1,2,\ldots ,q\right)$
**8**
Set the best position of the particle to $EE_{gb}^{\left(w\right)}=\max\left(EE_{1}^{\left(w\right)},EE_{2}^{\left(w\right)},\ldots ,EE_{q}^{\left(w\right)}\right)$
**9**
**Set** the global best particle as $gb^{\left(w\right)}=z_{U}^{\left(w\right)}$, where $U=\underset{k=\left\{1,2,\ldots ,q\right\}}{\arg\;\max}\left\{\left(EE_{k}^{\left(w\right)}\right)\right\}$
**10**
Increase the number of iterations w=w+1
**11**
From Equation (29), renew the velocities of the particles
**12**
From Equation (31) renew the locations of the particles
**13**
Calculate new EE of each particle: $EE_{gb}^{\left(w\right)}$
**14**
Get the best location of the renewed EE: $EE_{gb}^{\left(w\right)}=\max\left(EE_{1}^{\left(w\right)},EE_{2}^{\left(w\right)},\ldots ,EE_{q}^{\left(w\right)}\right)$
**15**
Renew $pb_{k}^{\left(w\right)}$ and $gb^{\left(w\right)}$
**16**
**if**$\left(\frac{EE_{gb}^{\left(w-1\right)}-EE_{gb}^{\left(w\right)}}{EE_{gb}^{\left(w\right)}}>\xi \right)$ and $\left(w<I_{T}\right)$
**17**
Go to step 21
**18**

**else**

**19**
Go to step 10
**20**

**end if**

**21**

**End**

**22**
**Return** the best solution of the PA subproblem

## 5. Simulation Results

In this section, we present simulation results to illustrate the benefits of the proposed RS and PA approaches to maximizing the EE of a network. Unless stated otherwise, simulation parameters for the proposed PSO algorithm are presented in Table 3. The values of the simulation parameters of the considered system model are listed in Table 4 which had already been adopted in [5,9]. We use the coordinates of the users (in meters) to describe the node locations. To show the effectiveness of the proposed scheme, we divided the simulation results into two groups because we developed two sub-problems: one for the RS and one for the PA. Therefore, the simulation results are also presented by separating them into two cases. In the first case, we compared the proposed ANFIS architecture-based RS scheme with exhaustive search-based RS schemes from [15,16], OpportunisticRS scheme, and RandomRS scheme to reflect the effectiveness of the proposed ANFIS-based RS scheme. In this simulation, we used the PSO-based PA scheme to allocate transmission power to D2D users of all the compared schemes. In the second case, we compared the proposed PSO-based PA scheme with the exhaustive search–based PA scheme from [15,16], equal power allocation (EPA) scheme, and random PA scheme with considering ANFIS architecture as a RS scheme to show the EE performance. In the RandomRS and RandomPA schemes the D2D relay is selected randomly without following any rules which can significantly degrade the joint received SINR at the D2D destination and the SINR at the base station. In the OpportunisticRS scheme, the D2D source selects the surrounding D2D relay based on full information about the CSI in the network. The D2D RS criterion of the OpportunisticRS scheme was defined in [24,25], as

(32) $$i^{*}=\underset{\;i=\left(1,2,3,\ldots ,L\right)}{argmaxmin}\left\{{\left|h_{SR_{i}}\right|}^{2},{\left|h_{R_{i}D}\right|}^{2}\right\}$$

**Figure 10:**
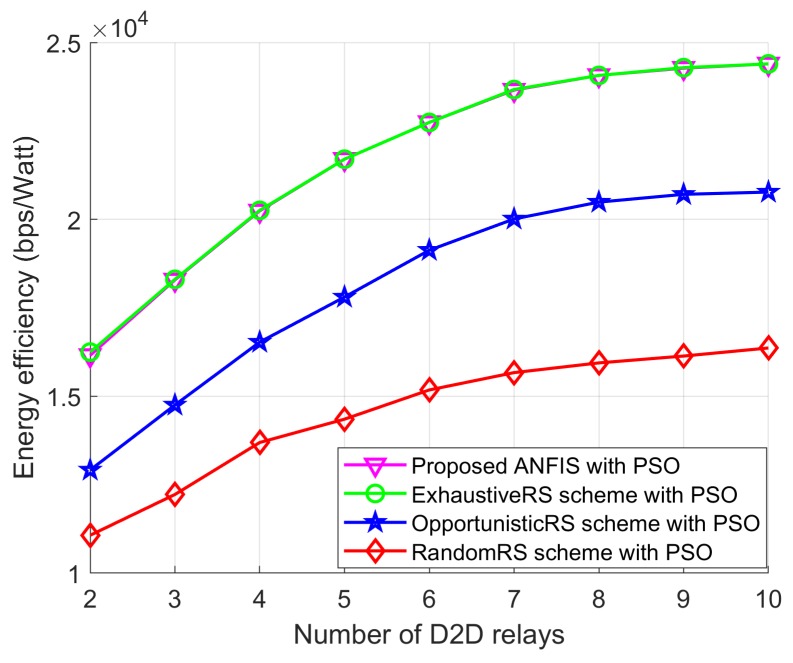
EE performance with *L*.

**Figure 11:**
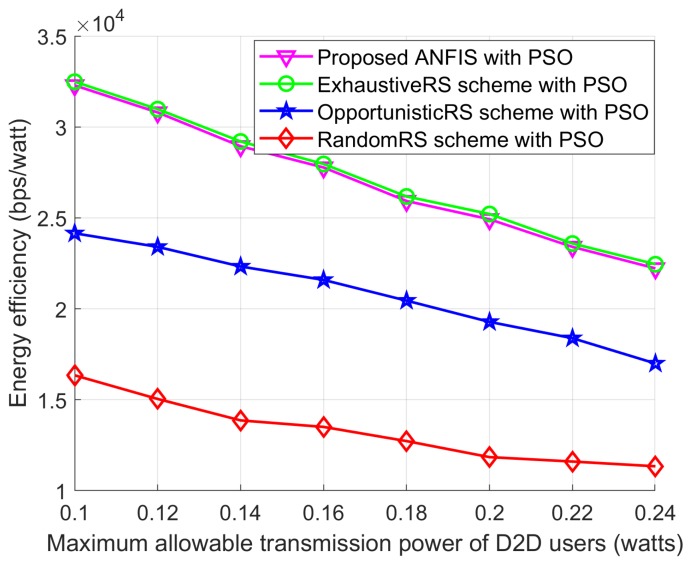
EE performance with *P_Max_*.

**Figure 12:**
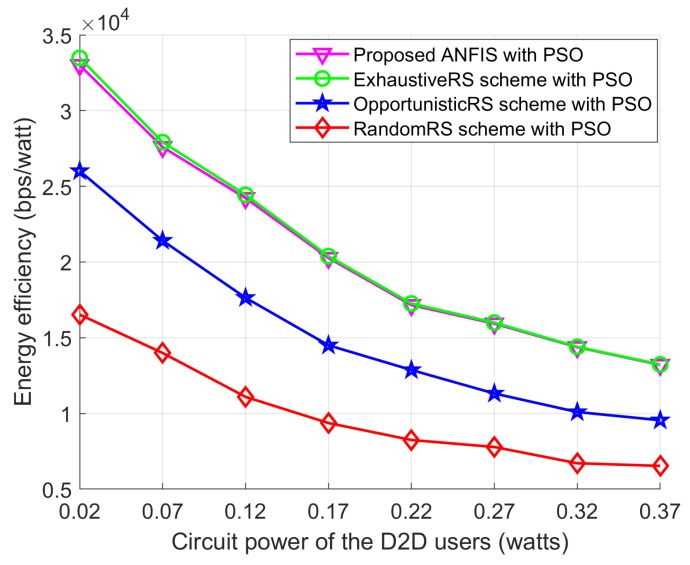
EE performance with *P_Cir_*.

We considered a cellular network with a radius of 300 m. The BS, the D2D destination, and the cellular user were fixed in their own positions, and the D2D relays were randomly distributed within transmission range of the D2D source. Without loss of generality, other network parameters were fixed as shown in Table 3 and Table 4. We compared the EE performance with the following schemes: (1) the proposed ANFIS with PSO: the proposed RS algorithm for EE maximization with PSO; (2) the ExhaustiveRS scheme with PSO: search all possible D2D relays, and select the best D2D relay that shows maximum EE, and then execute the proposed PSO to allocate transmission power to the D2D relay; (3) the OpportunisticRS scheme with PSO: select the D2D relay based on the rule in (32), and allocate power to the selected D2D relay via the proposed PSO algorithm; and (4) the RandomRS scheme with PSO: randomly select a D2D relay within transmission range of the D2D source, and use the PSO algorithm to assign transmission power to the selected relay. In Figure 10, Figure 11 and Figure 12 we show the performance of the proposed scheme according to the effectiveness of RS scheme where the positions of the cellular user (CU), the D2D source, and the D2D destination are fixed as listed in Table 4. The D2D relays are randomly distributed within the transmission range of the D2D source. In the simulation results, we consider single cellular user scenario where a D2D source sends its own information to the D2D destination by the aid of selected D2D relay. The following simulations results are obtained by averaging Monte Carlo trials. In each trials, the channel conditions are independently determined. The cases of different time-varying fading channel in [26] and a multi-user scenario for RS with an arbitrary number of sources in the networks with a Poisson field of nodes in [27] can be tested with additionally studying the channel estimation and the RS, but it is out of scope in this paper.

Figure 10 shows the EE performance of RA-D2D communications achieved by all the above schemes versus the number of D2D relays. We can see that the proposed ANFIS with the PSO scheme provides as close-to-optimal performance as the ExhaustiveRS scheme with the PSO scheme, which validates the effectiveness of our proposed ANFIS-based RS scheme. In addition, the EE achieved by the proposed ANFIS with PSO shows better performance than the OpportunisticRS scheme with PSO and the RandomRS scheme with PSO. The achieved EE from all schemes first increases, and then approaches a constant value, even if the number of D2D relays is increased. This is because of the maximum permissible transmission power for D2D users. The EE performance of all schemes remains flat, even though the number of D2D relays increases, because the transmission power assignment to the selected D2D relay reaches the maximum permissible transmission power limit. In addition, we also see that the OpportunisticRS scheme with PSO outperforms the RandomRS scheme with PSO. In RandomRS scheme with PSO, the D2D relay is selected without following any rules which can significantly degrade the joint received SINR at the D2D destination and the SINR at the base station. On the other hand, the proposed scheme selects the best D2D relay with having the maximum received SINR at the D2D destination and the SINR at the base station. Therefore, the EE performance of random scheme is always far from the proposed scheme.

Figure 11 shows the EE performance according to the maximum permissible transmission power for D2D users of the proposed ANFIS with PSO and the other schemes. As seen in Figure 11, the EE of all schemes drops as the maximum allowable transmission power of the D2D users increases. This is because of the total power consumption used to calculate the EE with D2D communications. When the transmission power increases, the total power consumption also increases, and it decreases the EE in D2D communications. The proposed ANFIS with PSO can reach close-to-optimal EE performance, along with the ExhaustiveRS scheme with PSO, and it shows better performance than the two other schemes. Owing to the RS impact of the proposed ANFIS architecture, the proposed ANFIS with PSO shows better EE performance than the other two schemes.

Figure 12 shows the effect of the circuit power for D2D users on EE performance under all the schemes. Specifically, the EE obtained by all schemes decreases as the circuit power of the D2D users increases, since the circuit power is always disadvantageous to the EE calculation. The proposed ANFIS scheme with PSO offers EE performance very close to the ExhaustiveRS scheme with PSO, which illustrates the effectiveness of the proposed ANFIS architecture for D2D RS in Section 4. In addition, the performance gap between the OpportunisticRS scheme with PSO and the RandomRS scheme with PSO is due the randomness of selecting the D2D relay in the networks. This observation of EE performance versus the circuit power of the D2D users demonstrates the practical interest in designing energy-efficient, oriented RA algorithms.

**Figure 13:**
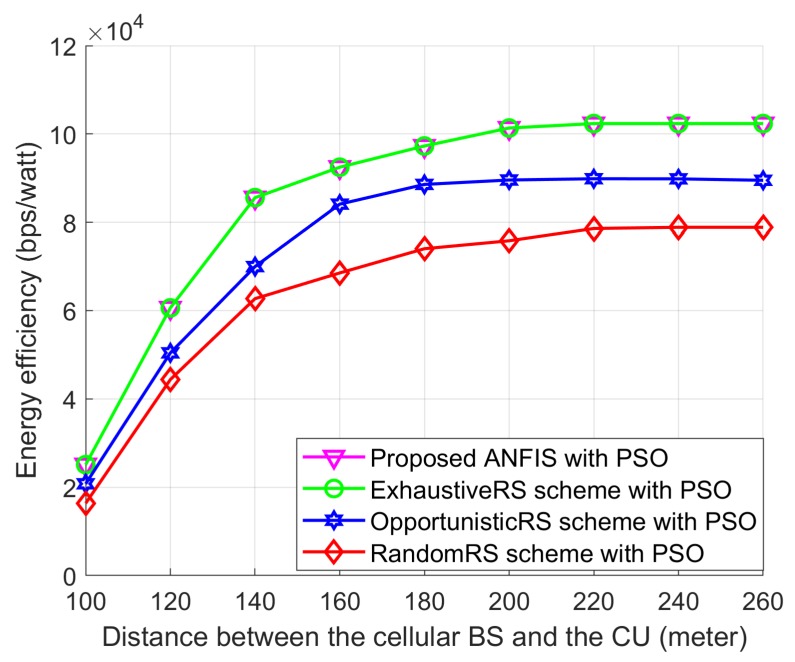
Effect of the relay-selection schemes on energy efficiency according to the distance between the cellular BS and the CU when the proposed PSO is used in all relay-selection schemes.

Figure 13 shows the EE performances of Proposed ANFIS with PSO, ExhaustiveRS scheme with PSO, OpportunisticRS scheme with PSO, and RandomRS scheme with PSO for different locations of cellular user. In this simulation, in order to observe the effect of the distance between the cellular BS and the CU on relay selection schemes, we assume that the proposed PSO scheme is used in all relay-selection schemes. In addition, the D2D source and the D2D destination are fixed on their positions and the D2D relays are randomly deployed within the transmission range of the D2D source and we change the location of the cellular user (CU) such that the distance between the cellular BS and the CU can be changed from 100 m to 260 m. Figure 13 shows that the EE performance of RA-D2D communications is improved when the distance between the BS and the CU increases. The reason is that the interference by the CU to the D2D relay and the D2D destination is decreased as the CU is moving far away from the D2D users. Also, Figure 13 shows that the proposed ANFIS with PSO provides close-to-optimal performance as the ExhaustiveRS scheme with PSO, which validates the effectiveness of the proposed ANFIS-based RS scheme. The EE performance of all schemes first increases and approaches a constant value, even though the distance between the cellular BS and the CU increases, which is mainly due to the fact that the transmission power assignment to the D2D users reaches their maximum power limits.

**Figure 14:**
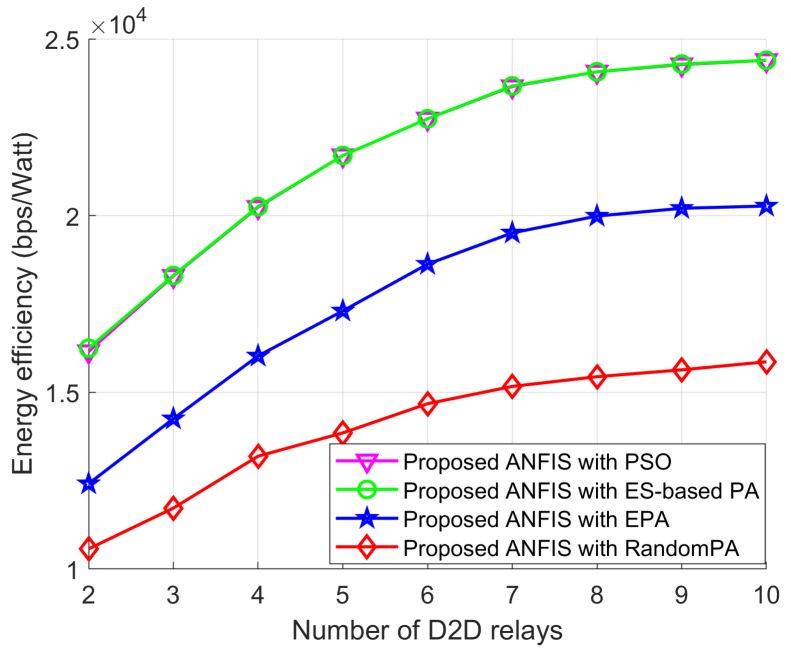
EE performance with *L*.

**Figure 15:**
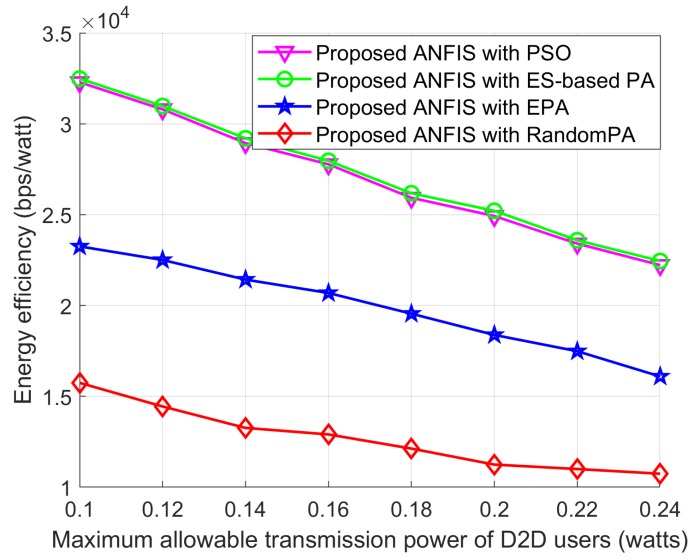
EE performance with *P_Max_*.

**Figure 16:**
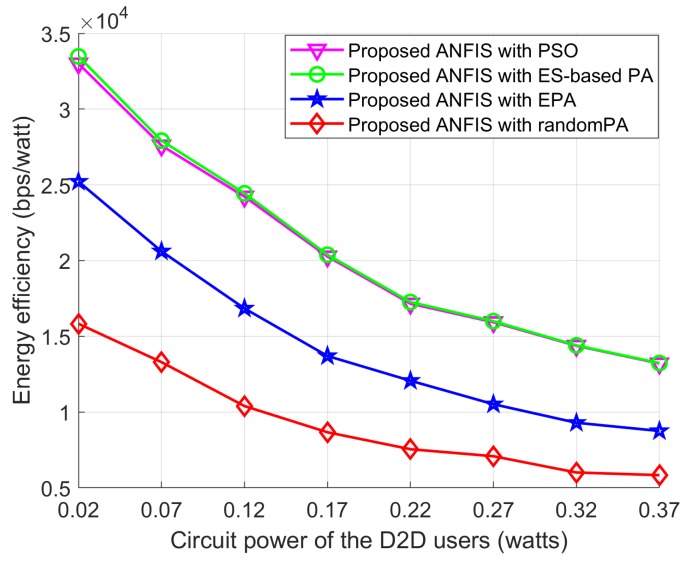
EE performance with *P_Cir_*.

In Figure 14, Figure 15 and Figure 16 we show the performance of the proposed scheme according to the effectiveness of PA scheme where the positions of the cellular user (CU), the D2D source, and the D2D destination are fixed as listed in Table 4. The D2D relays are randomly distributed within the transmission range of the D2D source. For all schemes, the proposed ANFIS architecture is commonly used for RS, but PA scheme for the D2D source and relay is different: (1) PSO PA: allocate transmission power by using the proposed PSO algorithm; (2) ES-based PA: exhaustively searching the optimal power in the given power space; (3) EPA: assign transmission power equally; (4) RandomPA: assigns transmission power randomly.

Figure 14 shows the EE performance according to the number of D2D relays for all schemes. PSO PA scheme shows better EE performance than EPA and RandomPA schemes because the PSO algorithm can provide sub-optimality. In addition, PSO PA scheme shows close optimal EE performance to ES-based PA scheme. The EE performance of all schemes remain constant after certain number of relays because of the restriction of the permissible transmission power.

Figure 15 shows the EE performance according to maximum allowable transmission power of the D2D users. As seen in Figure 15, the EE performance of all schemes decrease as the maximum allowable transmission power of the D2D users increases. This is because of the total transmission power consumption of the networks as discussed above. PSO PA scheme shows more improved EE performance than other two schemes. Moreover, PSO PA scheme can reach close optimal EE performance to ES-based PA scheme.

Figure 16 shows the EE performance according to circuit power of the D2D users. As can be seen from Figure 16 that the EE performance of all schemes decreased as the circuit power of D2D users increase. This is because the circuit power of the D2D users has a dominant effect on increasing the total power consumption by the networks. As is known, the EE performance metric used in this paper is the ratio between the data rate and the total transmission power consumption of the networks, therefore, with the increasing of circuit power the EE performance is always decreasing. The Proposed ANFIS with PSO shows superior EE performance than the other two schemes and it can reach close optimal EE performance to the Proposed ANFIS with ES-based PA scheme.

**Figure 17:**
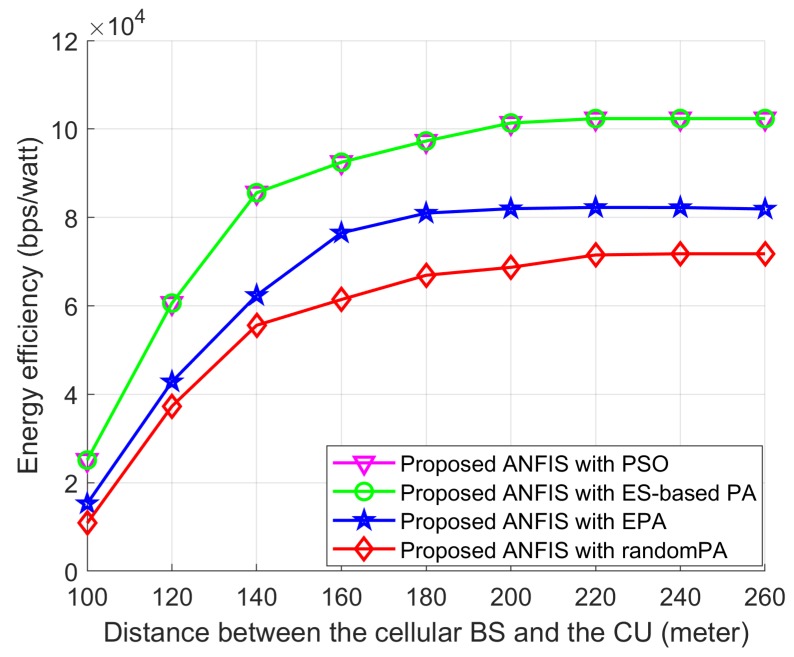
Effect of the power allocation schemes on energy efficiency according to the distance between the cellular BS and the CU when the proposed relay-selection, ANFIS, is used in all powerallocation schemes.

Similarly, in order to observe the effect of the distance between the cellular BS and the CU on the power allocation schemes, we assume that the proposed ANFIS scheme is used in all power-selection schemes. In addition, the D2D source and the D2D destination are fixed on their positions and the D2D relays are randomly deployed within the transmission range of the D2D source. Figure 17 shows that the EE performance of RA-D2D communications is improved when the distance between the BS and the CU increases. The reason is that the interference by the CU to the D2D relay and the D2D destination is decreased as the CU is moving far away from the D2D users. Also, Figure 17 shows that Proposed ANFIS with PSO provides close-to-optimal performance as the Proposed ANFIS with ES-based PA, which validates the effectiveness of the proposed PSO-based PA scheme. The EE performance of all schemes first increases and approaches a constant value, even though the distance between the cellular BS and the CU increases, which is mainly due to the fact that the transmission power assignment to the D2D users reaches their maximum power limits.

## 6. Conclusions

In this paper, we have investigated relay-assisted D2D communication in 5G wireless networks. Specifically, D2D RS and PA to the selected D2D relay have been jointly optimized by utilizing ANFIS architecture and PSO algorithm to enhance the EE performance of D2D communications while guaranteeing the maximum permissible transmission power of the D2D uses and the minimum data rate requirements of the underlying cellular user. Simulation results have shown that the proposed scheme with ANFIS and PSO attains close-to-optimal performance to the exhaustive search-based D2D relay selection PA schemes. Moreover, the EE performance achieved by the proposed scheme is much higher than some conventional schemes. The information transmission between the D2D source and the D2D destination is vulnerable when eavesdroppers coexist in the networks. Moreover, the resource block allocation with different time-varying fading channels and a multi-user scenario for RS with an arbitrary number of sources in the networks are also beyond the scope of this paper. Therefore, security issues of RA-D2D communications in 5G networks, RB allocation with different time-varying fading channels, and multi-user scenario for RS and PA with an arbitrary number of sources in the networks are left for our future work. 

## Figures and Tables

**Table 1:** Summary of main symbols.

Symbol	Description
*C* and *D*	Cellular user and the D2D source, respectively
$R_{i}$	*i*-th D2D relay, where $i=\left\{1,2,3,\ldots ,L\right\}$
$P_{S}$ and $P_{R_{i}}$	Transmission power of the D2D source and transmission power of the *i*-th D2D relay, respectively
$\gamma_{SR_{i}}$ and $\gamma_{R_{i}D}$	SINR between $S\to R_{i}$ and $R_{i}\to D$, respectively
$\gamma_{SR_{i}D}$ and $\gamma_{CB}$	Received SINR at the D2D destination and SINR between C→B, respectively
*D* and *L*	D2D destination and total number of D2D relays, respectively
$h_{SR_{i}}$ and $h_{R_{i}D}$	Channel coefficient between $S\to R_{i}$ and channel coefficient between $R_{i}\to D$ , respectively
$h_{CB}$ and $h_{CD}$	Channel coefficient between C→B and channel coefficient between C→D, respectively
${ℜ}_{SR_{i}D}$ and ${ℜ}_{CB}$	Data rate of RA-D2D communications, and data rate at the BS by the CU, respectively
$P_{Total}$	Total power consumption by the networks
*W*	Allocated bandwidth of the networks
$h_{CR_{i}}$	Channel coefficient between $C\to R_{i}$
$P_{Cir}$	Circuit power of the D2D source and the D2D relay
λ	Power amplifier efficiency
$N_{0}$	Noise variance
$\gamma_{R}^{Min}$	Minimum SINR requirement for the D2D relay
$P_{Max}$	Maximum allowable transmission power for the D2D UEs
$\gamma_{R}^{Min}$	Minimum SINR requirement for the D2D relay

**Table 2:** ANFIS structure information.

ANFIS Parameter Type	Value
Number of inputs	2
Membership type	Generalized bell-shaped MF
Number of memberships	3 × 3
Training data set	70%
Testing data set	30%
Number of nodes	35
Number of linear parameters	27
Number of nonlinear parameters	18
Total number of parameters	45

**Table 3:** Simulation parameters.

Parameter	Notation	Value
Number of particles	*P*	50
Scaling factors of PSO	$c_{1}$ and $c_{2}$	2
Maximum number of generations	$I_{T}$	200
Precision of PSO	ξ	10^−6^

**Table 4:** Simulation parameters.

Parameter	Notation	Value
Network size		300 × 300 m
Bandwidth	*W*	180 kHz
Minimum data rate requirement of CU users	${ℜ}_{C}^{Min}$	20 Kb/s
Minimum decodable SINR at the D2D relay	$\gamma_{R}^{Min}$	3 dB
Minimum decodable SINR at the D2D destination	$\gamma_{D}^{Min}$	3 dB
Noise spectral density	$N_{0}$	−174 dBm/Hz
Power amplifier efficiency	λ	0.38
Mobility		Static scenario
Number of D2D relays	*L*	10
Distance between D2D users		20 m
D2D source coordinates		(60, 0)
Cellular user base station coordinates		(50, 0)
Cellular user coordinates		(100, 10)
D2D destination coordinates		(80, 0)
Path gain for each link		0.097d3.76
